# A disappearing diabetes: extreme and reversible hyperglycemia during an ulcerative colitis flare

**DOI:** 10.1210/jcemcr/luag235

**Published:** 2026-09-11

**Authors:** Hitam Hagog Natour, Michal Kasher Meron, Pnina Rotman-Pikielny, Liat Barzilay-Yoseph

**Affiliations:** Institute of Endocrinology, Diabetes and Metabolism, Meir Medical Center, Kfar Saba 4428164, Israel; Institute of Endocrinology, Diabetes and Metabolism, Meir Medical Center, Kfar Saba 4428164, Israel; Gray Faculty of Medical and Health Sciences, Tel Aviv University, Tel Aviv, Israel; Institute of Endocrinology, Diabetes and Metabolism, Meir Medical Center, Kfar Saba 4428164, Israel; Gray Faculty of Medical and Health Sciences, Tel Aviv University, Tel Aviv, Israel; Institute of Endocrinology, Diabetes and Metabolism, Meir Medical Center, Kfar Saba 4428164, Israel

**Keywords:** stress hyperglycemia, ulcerative colitis, HbA1c, insulin resistance, reversible hyperglycemia

## Abstract

Stress hyperglycemia commonly occurs during acute illness; however, extreme hyperglycemia accompanied by markedly elevated glycated hemoglobin (HbA1c) in individuals without pre-existing diabetes is rare and may mimic new-onset diabetes. We describe a 56-year-old man with no prior history of diabetes who presented with severe diarrhea, abdominal pain, and weight loss and was found to have profound hyperglycemia, 603 mg/dL (SI: 33.5 mmol/L) (reference range, 70-99 mg/dL [SI: 3.9-5.5 mmol/L]), without metabolic acidosis. HbA1c was markedly elevated at 9.4% (reference range, 4.0%–5.7%), despite a normal value documented 2 months earlier. Evaluation demonstrated preserved endogenous insulin secretion, elevated homeostatic model assessment of insulin resistance, and negative autoimmune markers. Imaging excluded pancreatic pathology, while colonoscopy and histopathology confirmed active moderate ulcerative colitis (UC). The patient was treated with intravenous corticosteroids and insulin therapy, with rapid normalization of glycemia allowing discontinuation of insulin. Sustained euglycemia was observed after remission, with HbA1c normalizing to 4.7% at 3-month follow-up. This case highlights that severe inflammation during an UC flare can induce profound yet reversible hyperglycemia, emphasizing the need for careful interpretation and longitudinal follow-up.

## Introduction

Stress hyperglycemia is a transient elevation in blood glucose occurring in response to acute illness or physiological stress, independent of prior diabetic status. It is common in critically ill patients, including those with acute ischemic stroke and sepsis, and is associated with increased morbidity and mortality, particularly among individuals without pre-existing diabetes [1-3]. The pathophysiology involves a complex interplay of counter-regulatory hormones (catecholamines, cortisol), cytokines, and increased hepatic glucose production, resulting in insulin resistance and impaired glucose uptake [4]. Severe inflammatory conditions further amplify these responses. While the majority of the literature on stress hyperglycemia has focused on critically ill populations (eg, stroke, sepsis, acute pancreatitis, myocardial infarction), data regarding its occurrence and clinical relevance in inflammatory bowel disease, and ulcerative colitis (UC) in particular, remain limited [5-7].

In severe UC, both active inflammation and corticosteroid therapy may contribute to disturbances in glucose metabolism and the development of hyperglycemia, even in patients without pre-existing diabetes [4, 8-10]. Acute profound hyperglycemia with markedly elevated glycated hemoglobin (HbA1c) in patients with UC who do not have a prior diagnosis of diabetes is rare, and the literature specifically addressing this scenario is limited. Here, we report a striking case of profound, yet fully reversible hyperglycemia accompanied by a markedly elevated HbA1c in a patient with active UC and no prior personal history of diabetes.

This case illustrates the capacity of severe systemic inflammation to precipitate dramatic but transient disturbances in glucose homeostasis. It emphasizes the critical importance of careful clinical interpretation, longitudinal assessment, and follow-up to distinguish stress-related hyperglycemia from true new-onset diabetes, thereby preventing misdiagnosis and avoiding unnecessary long-term antidiabetic therapy. We contextualize this case within the existing literature.

## Case presentation

A 56-year-old overweight (body mass index 28 kg/m^2^) man with no prior history of diabetes, but a strong family history of type 2 diabetes, was admitted with profuse bloody and mucoid diarrhea (>10 episodes per day), abdominal pain, and an unintentional weight loss of 8 kg over the preceding 2 months. Despite frequent diarrhea, the patient maintained adequate oral fluid intake and did not exhibit clinical signs of significant dehydration on presentation. He was a nonsmoker, denied alcohol consumption, and had no significant past medical history other than overweight. He had no personal or family history of autoimmune disease, no recent medication exposure, and no symptoms suggestive of other endocrine disorders.

On admission, he was hemodynamically stable, with a blood pressure of 123/70 mm Hg, heart rate of 88 beats per minute, and temperature of 36.6°C. He appeared well and in no acute distress. Physical examination was notable only for mild diffuse abdominal tenderness without guarding, rebound, organomegaly, or palpable masses. Bowel sounds were normal. No peripheral edema, skin rash, or oral ulcers were observed.

## Diagnostic assessment

Initial laboratory evaluation demonstrated mild normocytic normochromic anemia (hemoglobin 11.7 g/dL [reference range, 13.5-17.5 g/dL]) and marked hyperglycemia, 603 mg/dL (SI: 33.5 mmol/L) (reference range, 70-99 mg/dL [SI: 3.9-5.5 mmol/L]). Arterial blood gas analysis showed no metabolic acidosis (pH 7.40 [reference range, 7.35-7.45], pCO_2_ 40 mm Hg [reference range, 35-45 mm Hg], HCO_3_^−^ 27.2 mmol/L [reference range, 21-28 mmol/L]), and findings were not consistent with a hyperosmolar hyperglycemic state. Liver and pancreatic enzyme levels were within normal limits, including aspartate aminotransferase (AST) 21 U/L (SI: 0.35 µkat/L; reference range, 7-37 U/L [SI: 0.12-0.62 µkat/L]), alanine aminotransferase (ALT) 39 U/L (SI: 0.65 µkat/L; reference range, 0-40 U/L [SI: 0-0.67 µkat/L]), amylase 47 U/L (SI: 0.78 µkat/L; reference range, 5-100 U/L [SI: 0.08-1.67 µkat/L]), and lipase 70 U/L (SI: 1.17 µkat/L; reference range, 13-60 U/L [SI: 0.22-1.00 µkat/L]). Serum calcium was within the normal range at 9.4 mg/dL (SI: 2.35 mmol/L) (reference range, 8.5-10.5 mg/dL [SI: 2.12-2.62 mmol/L]). Fecal elastase was normal (305 µg/g [reference range, >200 µg/g]), urinary amylase was mildly elevated (500 U/L [reference range, 0-460 U/L]), and celiac serology was negative. Inflammatory markers were not significantly elevated, including C-reactive protein (CRP) 1.2 mg/dL (SI: 12 mg/L; reference range, <0.5 mg/dL [SI: <5 mg/L]) and white blood cell count 8.3 × 10^3^/µL (reference range, 4.0-10.0 × 10^3^/µL). Additional laboratory data are presented in Table 1.

**Table 1:** Additional laboratory parameters

Parameter	Normal range	Two months preadmission	Admission	Three weeks postdischarge	Three months follow-up
Hemoglobin	13.5-17.5 g/dL (SI: 135-175 g/L)	14.5 g/dL (SI: 145 g/L)	11.9 g/dL (SI: 119 g/L)	12.8 g/dL (SI: 128 g/L)	13.7 g/dL (SI: 137 g/L)
WBC	4.0-10.0 × 10^3^/µL (SI: 4.0-10.0 × 10^9^/L)	6.4 × 10^3^/µL	8.3 × 10^3^/µL	8.9 × 10^3^/µL	9.1 × 10^3^/µL
Creatinine	0.7-1.3 mg/dL (SI: 62-115 µmol/L)	0.6 mg/dL (SI: 53 µmol/L)	0.6 mg/dL (SI: 53 µmol/L)	0.8 mg/dL (SI: 71 µmol/L)	0.7 mg/dL (SI: 62 µmol/L)
Sodium	135-145 mEq/L (SI: 135-145 mmol/L)	138 mEq/L	136 mEq/L	139 mEq/L	138 mEq/L
Potassium	3.5-5.1 mEq/L (SI: 3.5-5.1 mmol/L)	3.6 mEq/L	3.5 mEq/L	4.4 mEq/L	4.5 mEq/L
ALT	0-40 U/L (SI: 0-0.67 µkat/L)	19 U/L (SI: 0.32 µkat/L)	39 U/L (SI: 0.65 µkat/L)	25 U/L (SI: 0.42 µkat/L)	18 U/L (SI: 0.30 µkat/L)
AST	7-37 U/L (SI: 0.12-0.62 µkat/L)	16 U/L (SI: 0.27 µkat/L)	21 U/L (SI: 0.35 µkat/L)	21 U/L (SI: 0.35 µkat/L)	13 U/L (SI: 0.22 µkat/L)
GGT	7-49 U/L (SI: 0.12-0.82 µkat/L)	30 U/L (SI: 0.50 µkat/L)	28 U/L (SI: 0.47 µkat/L)	24 U/L (SI: 0.40 µkat/L)	32 U/L (SI: 0.53 µkat/L)
Total bilirubin	0.2-1.5 mg/dL (SI: 3.4-25.7 µmol/L)	0.8 mg/dL (SI: 13.7 µmol/L)	1.1 mg/dL (SI: 18.8 µmol/L)	1.1 mg/dL (SI: 18.8 µmol/L)	0.4 mg/dL (SI: 6.8 µmol/L)
LDH	120-250 U/L (SI: 2.0-4.2 µkat/L)	127 U/L (SI: 2.12 µkat/L)	145 U/L (SI: 2.42 µkat/L)	223 U/L (SI: 3.72 µkat/L)	124 U/L (SI: 2.07 µkat/L)
Amylase	5-100 U/L (SI: 0.08-1.67 µkat/L)	62 U/L (SI: 1.03 µkat/L)	47 U/L (SI: 0.78 µkat/L)	47 U/L (SI: 0.78 µkat/L)	50 U/L (SI: 0.83 µkat/L)
Lipase	13-60 U/L (SI: 0.22-1.00 µkat/L)	ND	70 U/L (SI: 1.17 µkat/L)	ND	ND
Urine amylase	0-460 U/L (SI: 0-7.67 µkat/L)	ND	500 U/L (SI: 8.35 µkat/L)	ND	ND
Fecal elastase	>200 µg/g	ND	305 µg/g	ND	ND
CRP	<0.5 mg/dL (SI: <47.6 nmol/L)	ND	1.2 mg/dL (SI: 114.3 nmol/L)	ND	ND
TSH	0.2-4.2 mU/L (SI: 0.2-4.2 mIU/L)	ND	0.81 mU/L	ND	ND
IgG4	3.9-86.4 mg/dL (SI: 0.039-0.864 g/L)	ND	54.8 mg/dL (SI: 0.548 g/L)	ND	ND

Reference ranges may vary slightly depending on laboratory standards. SI values are provided only where they differ from the corresponding conventional values.

Abbreviations: ALT, alanine aminotransferase; AST, aspartate aminotransferase; CRP, C-reactive protein; GGT, gamma-glutamyl transferase; IgG4, immunoglobulin G subclass 4; LDH, lactate dehydrogenase; ND, no data; TSH, thyroid-stimulating hormone; WBC, white blood cell count.

HbA1c was markedly elevated at 9.4% (reference range, 4.0%–5.7%), compared with a normal value of 5.08% obtained 2 months earlier during routine health screening prompted by overweight and a strong family history of type 2 diabetes (Table 2). Fasting insulin was 7.0 µIU/mL (reference range, 2.6-25.0 µIU/mL) and C-peptide 1.6 ng/mL (reference range, 0.5-2.0 ng/mL); the homeostatic model assessment of insulin resistance (HOMA-IR) was 10.4. Autoimmune markers for diabetes, including anti–glutamic acid decarboxylase and IA-2 antibodies, were negative (Table 3).

**Table 2:** Glycemic parameters over time

Parameter	Normal range	Two months preadmission	Admission	Three months follow-up
Glucose (mg/dL)	70-99 (SI: 3.9-5.5 mmol/L)	95 (SI: 5.3 mmol/L)	603 (SI: 33.5 mmol/L)	85 (SI: 4.7 mmol/L)
HbA1c (%)	4.0-5.7	5.08	9.4	4.7

Reference ranges may vary slightly depending on laboratory standards.

Abbreviations: HbA1c, glycated hemoglobin.

**Table 3:** Evaluation for diabetes type at presentation

Test	Result	Normal range
C-peptide, ng/mL (SI: nmol/L)	1.6 ng/mL (SI: 0.53 nmol/L)	0.5-2.0 ng/mL (SI: 0.17-0.66 nmol/L)
Insulin, µIU/mL (SI: pmol/L)	7.0 µIU/mL (SI: 48.6 pmol/L)	2.6-25.0 µIU/mL (SI: 18.1-173.6 pmol/L)
HOMA-IR	10.4	ND
Anti-GAD antibodies	Negative	Negative
IA-2 antibodies	Negative	Negative
pH	7.44	7.35-7.45
HCO_3_^−^, mmol/L	27.2 mmol/L	21-28 mmol/L

Reference ranges may vary slightly depending on laboratory standards.

Abbreviations: GAD, glutamic acid decarboxylase; HOMA-IR, homeostatic model assessment of insulin resistance; IA-2, insulinoma-associated antigen-2; ND, no data.

**Figure 1:**
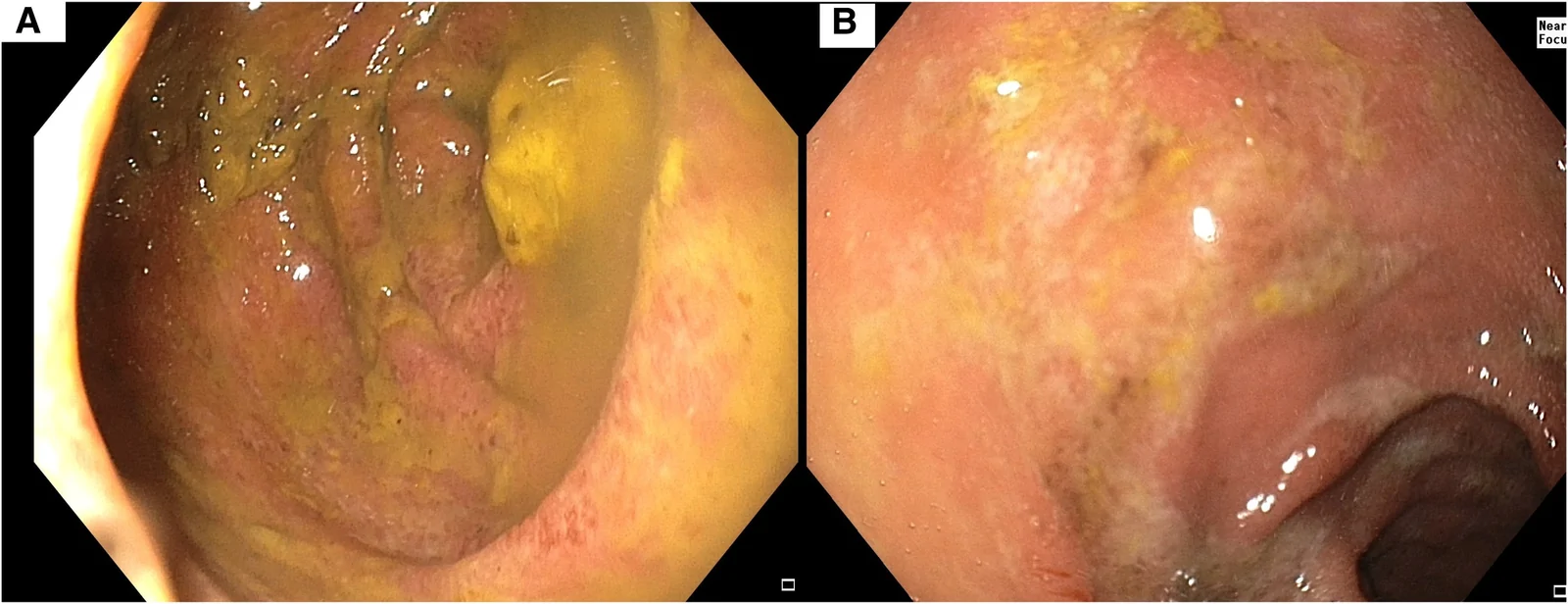
Colonoscopic findings. Endoscopic evaluation of the colon during the acute flare of ulcerative colitis. (A) Diffuse mucosal erythema, edema, and loss of vascular pattern with adherent yellow exudates. (B) Continuous inflammation with friability and superficial ulcerations consistent with active colitis.

**Figure 2:**
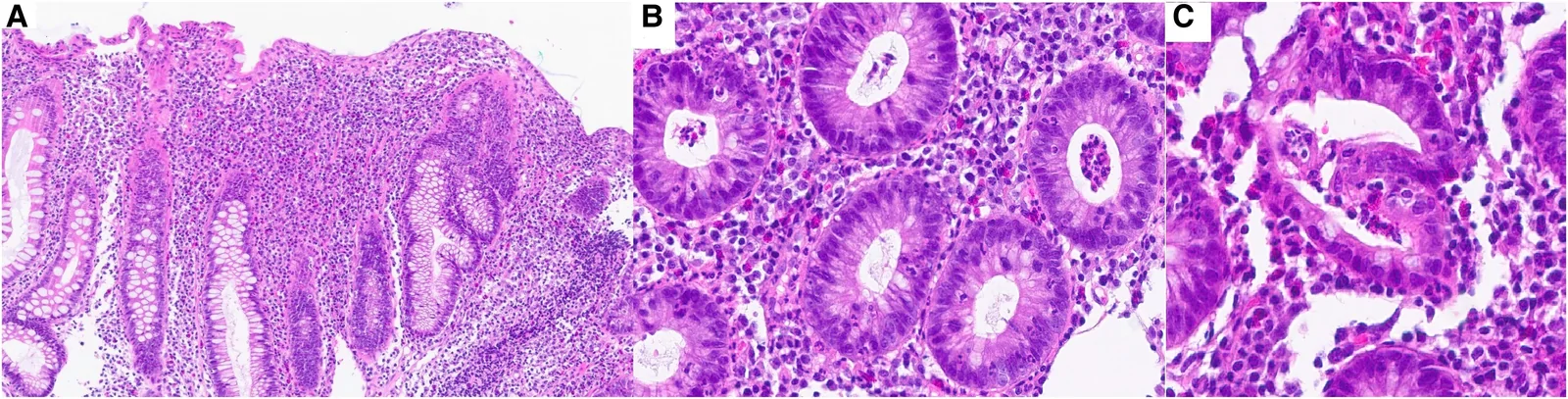
Colonic histopathology. Hematoxylin and eosin–stained colonic biopsy specimens demonstrate features of active chronic colitis consistent with ulcerative colitis. (A) Low-power view showing diffuse inflammatory infiltrate in the lamina propria with crypt architectural distortion and surface mucosal irregularity (original magnification ×100). (B) Higher-power view highlighting cryptitis with neutrophils within crypt lumina (original magnification ×400). (C) Higher-power view demonstrating dense inflammatory infiltrate in the lamina propria composed of lymphocytes, plasma cells, and neutrophils, with focal epithelial injury (original magnification ×400).

Abdominal computed tomography performed as part of the evaluation for new-onset diabetes demonstrated mild pancreatic haziness without focal lesions and bowel wall thickening suggestive of colitis. Infectious workup, including blood cultures and human immunodeficiency virus testing, was negative. Rheumatologic and autoimmune evaluation was unremarkable. Colonoscopy revealed findings consistent with inflammatory bowel disease (Fig. 1), and histopathological examination of colonic biopsies confirmed a new diagnosis of active moderate UC (Fig. 2).

## Treatment

The patient was initiated on a basal–bolus insulin regimen consisting of insulin glargine and preprandial insulin lispro (Humalog). Following the diagnosis of UC, intravenous hydrocortisone (100 mg 3 times daily) was started, along with sulfasalazine. During hospitalization, glucose levels were monitored with frequent capillary blood glucose measurements. The patient was discharged after 7 days of hospitalization on insulin therapy and corticosteroids. Upon discharge, continuous glucose monitoring (CGM) was initiated for outpatient glucose monitoring.

## Outcome and follow-up

Three weeks after discharge, CGM demonstrated predominantly normal glucose values. The patient reported one episode of mild hypoglycemia while receiving insulin therapy, which resolved promptly following oral glucose administration, with no subsequent hypoglycemic events. Given sustained normoglycemia and the absence of recurrent hyperglycemia, insulin therapy was formally discontinued. With treatment and tapering of corticosteroids, gastrointestinal symptoms resolved. At 3-month follow-up, glucose levels remained within the normal range (85-110 mg/dL), and HbA1c normalized to 4.7%.

The patient remained off insulin therapy and was scheduled for regular follow-up every 3 months. He was advised to maintain a healthy lifestyle and to monitor glucose levels during future episodes of acute illness or physiological stress.

## Discussion

Stress hyperglycemia is a well-recognized response to acute illness and most often reflects unmasking of underlying or previously undiagnosed diabetes rather than a fully reversible condition [1-4]. In contrast, the present case describes an unusual presentation of extreme hyperglycemia with markedly elevated HbA1c in a patient without prior diabetes, followed by complete normalization after resolution of an UC flare.

In patients with UC, dysglycemia is commonly attributed to corticosteroid therapy or occurs in those with pre-existing metabolic risk factors [4, 8-10]. In the present case, however, severe hyperglycemia was documented before exposure to corticosteroids, making steroid-induced hyperglycemia an unlikely primary explanation. Although systemic inflammatory markers were only mildly elevated, active UC is characterized by significant mucosal inflammation that may not be fully reflected by circulating biomarkers such as CRP. Indeed, up to 20-25% of patients with active UC may have normal or only minimally elevated CRP despite substantial endoscopic disease activity [11, 12].

One potential mechanism involves increased intestinal permeability during active disease, leading to translocation of bacterial lipopolysaccharides and activation of inflammatory pathways such as toll-like receptor 4 (TLR4), which have been associated with impaired glucose homeostasis and insulin resistance in experimental and clinical studies [13]. In the present case, the markedly elevated HOMA-IR of 10.4 is consistent with marked insulin resistance during the acute inflammatory phase. Together with the absence of pre-existing diabetes, preserved endogenous insulin secretion, and complete resolution of hyperglycemia following remission of UC, these findings support inflammation-induced insulin resistance as a major contributor to the observed metabolic disturbance. Nevertheless, the precise pathophysiological mechanisms underlying this phenomenon are likely multifactorial and remain incompletely understood.

To place our findings in context, several studies have examined the relationship between inflammatory bowel disease and abnormalities in glucose metabolism, although reports of extreme reversible hyperglycemia remain scarce. A comparison of these previously published studies with the present case is summarized in Table 4. Redsted et al demonstrated increased insulin resistance and elevated fasting glucose levels in patients with acute severe UC, with improvement following disease remission. Similarly, Bergemalm et al reported reversible disturbances in glucose metabolism and insulin sensitivity that normalized after achieving steroid-free remission; however, patients in both studies had received corticosteroid therapy as part of the management of the acute flare. These observations support the concept that active intestinal inflammation is associated with insulin resistance and disturbed glucose metabolism. In contrast, our patient developed profound hyperglycemia before corticosteroid therapy was initiated, allowing a clearer distinction between inflammation-associated hyperglycemia and glucocorticoid-induced hyperglycemia. McDonnell et al focused primarily on glucocorticoid-induced hyperglycemia in hospitalized patients with inflammatory bowel disease. Finally, Kang et al demonstrated an increased long-term risk of diabetes among patients with inflammatory bowel disease in a large population-based cohort, emphasizing the importance of continued metabolic follow-up even after normalization of glycemia [8, 9, 14, 15].

**Table 4:** Hyperglycemia in ulcerative colitis and IBD: comparison with the present case

Study	Year	Population	Primary endpoint	Pre-existing diabetes	Steroid exposure at assessment	HbA1c at presentation	Glycemic pattern	Reversibility
Redsted et al [14]	2024	22 patients with acute severe UC	Insulin resistance (HOMA-IR) during flare	Not specified	Yes (≥2 days)	Not reported	↑ HOMA-IR and fasting glucose	Improved after discharge
Bergemalm et al [8]	2025	5 adults with moderate–severe UC	Change in insulin sensitivity during flare	No diabetes	Yes (IV glucocorticoids ≥2 days)	Not reported	Marked insulin resistance	Normalized in steroid-free remission
McDonnell et al [9]	2020	94 hospitalized IBD patients	Incidence of glucocorticoid-induced hyperglycemia	With and without diabetes	Yes (all received IV steroids)	Not systematically reported	Steroid-associated hyperglycemia	Variable; steroid-dependent
Kang et al [15]	2019	8070 IBD patients vs controls	Incident diabetes during longitudinal follow-up	Excluded baseline diabetes	Adjusted for steroid exposure	Not reported	Increased long-term diabetes risk in IBD; stronger association in Crohn disease than UC	Not applicable
Present Case	2026	Adult with severe UC flare	Extreme hyperglycemia mimicking new-onset diabetes during flare	No known diabetes; normal HbA1c 2 months prior	No steroid exposure at presentation	Markedly elevated (diabetic range)	Severe hyperglycemia disproportionate to prior glycemic status	Complete normalization after inflammatory remission

Abbreviations: HbA1c, glycated hemoglobin; HOMA-IR, homeostatic model assessment of insulin resistance; IBD, inflammatory bowel disease; IV, intravenous; UC, ulcerative colitis.

Our case differs from previous reports in several important aspects. First, the patient had no prior diagnosis of diabetes and a normal HbA1c documented only 2 months before presentation. Second, profound hyperglycemia was already present before exposure to corticosteroid therapy, making steroid-induced hyperglycemia an unlikely primary explanation. Finally, complete normalization of both hyperglycemia and HbA1c following remission of UC supports a transient disturbance in glucose homeostasis associated with severe inflammation rather than established diabetes.

Preserved endogenous insulin secretion, negative autoimmune markers, and complete resolution of hyperglycemia further support this interpretation. Although HbA1c remains an essential diagnostic tool, it should be interpreted cautiously during acute inflammatory illness, as it reflects weighted glycemic exposure over the preceding weeks and may be influenced by acute metabolic disturbances and altered erythrocyte turnover [16]. The previously documented normal HbA1c further supports the absence of chronic dysglycemia.

Although stress hyperglycemia is generally transient, it has been associated with an increased long-term risk of developing diabetes in previously nondiabetic individuals [2, 6, 7]. Therefore, patients presenting with severe stress hyperglycemia should undergo careful longitudinal metabolic follow-up, even after complete clinical recovery.

Severe stress hyperglycemia may occur during acute inflammatory states in the absence of underlying diabetes and may completely resolve following treatment of the precipitating condition. This case highlights the importance of integrating clinical history, laboratory findings, and longitudinal follow-up when interpreting marked hyperglycemia and HbA1c during acute illness. Further studies are needed to better define the mechanisms and long-term metabolic implications of inflammation-associated stress hyperglycemia.

## Learning points

- Severe stress hyperglycemia may occur during active UC, even in individuals without pre-existing diabetes.
- Markedly elevated HbA1c during acute inflammatory illness may not necessarily reflect chronic glycemic status and should be interpreted in the appropriate clinical context.
- Preserved endogenous insulin secretion, negative autoimmune markers, and subsequent normalization of glycemia may help distinguish transient stress hyperglycemia from newly diagnosed diabetes.
- Active inflammation may induce marked insulin resistance, contributing to profound but reversible hyperglycemia.
- Longitudinal metabolic follow-up is essential, as patients with stress hyperglycemia may remain at increased risk of future diabetes despite complete clinical recovery.

## Data Availability

Data sharing is not applicable to this article as no datasets were generated or analyzed.
